# Small gastrointestinal stromal tumour of the duodenum causing a life-threatening bleeding - A case report and review of the literature

**DOI:** 10.1016/j.ijscr.2019.03.035

**Published:** 2019-03-30

**Authors:** Milena Taskovska, Mirko Omejc, Jan Grosek

**Affiliations:** a Department of Urology, University Medical Centre Ljubljana, Zaloška c. 7, 1000 Ljubljana, Slovenia; b Department of Abdominal surgery, University Medical Centre Ljubljana, Zaloška c. 7, 1000 Ljubljana, Slovenia

**Keywords:** Gastrointestinal stromal tumor, Duodenum, Bleeding, Surgery, Case report

## Abstract

**Introduction:**

Duodenal gastrointestinal stromal tumors (DGISTs) are mesenchymal tumors. Main presenting symptoms are gastrointestinal bleeding and non-specific abdominal pain. Surgical treatment is golden standard for treatment of DGISTs. Tumour location and size determine the surgical strategy. Recurrence rate depends on tumour location and completeness on removal.

**Presentation of case:**

75 years old male fell ill with hematemesis, hemohesia and dizziness. Oesophagogastroduodenoscopy (EGD) did not reveal the source of bleeding. Upon admission, a second EGD was performed due to loss of consciousness and anaemia, which revealed only approximate source of bleeding in the third portion of the duodenum. The exact bleeding spot could not be reached and bleeding could not be controlled endoscopically. Patient was transferred to the operating room for an emergency explorative laparotomy. After extensive mobilisation of the duodenum a 2 × 2 cm intraluminal tumour with central bleeding ulceration was found in D3. The diseased part of the duodenum was excised and the duodenotomy was closed with interrupted sutures. Final pathohistological report confirmed gastrointestinal stromal tumour, pT1. Postoperative period was uneventful.

**Discussion:**

Gastrointestinal stromal tumours are most common in males aged 60–65 years. Surgical treatment is a golden standard. Surgical approach depends upon tumor location and size. In case of major bleeding surgical procedure is lifesaving and diagnostics for staging purposes are omitted.

**Conclusion:**

Gastrointestinal stromal tumors are a potential source of life-threatening bleeding. In such cases an emergency operation is often indicated with aim to remove the tumour en bloc and achieve hemostasis as soon as possible.

## Highlights

• Gastrointestinal stromal tumours could be source of massive (life-threatening bleeding).
• If massive bleeding is present in patient with gastrointestinal stromal tumour, surgical treatment is indicated, without further diagnostics.
• Depending on location and size of GIST, extend of surgical treatment varies. En bloc resection is required, role of lymphadenectomy is not clear.
• In setting when patient is stable, diagnostic evaluation is performed first, to access size, location of tumour and plan best surgical approach.
• Treatment of gastrointestinal stromal tumours should be multidisciplinary.

## 1 Introduction

Gastrointestinal stromal tumors (GISTs) are the most common mesenchymal tumors. They can originate from the entire gastrointestinal tract, most commonly from stomach (60%), small intestine (20–30%) and duodenum (5%). About 10–30% of all neoplasms of duodenum are duodenal GISTs (DGISTs) [1]. Most frequent symptoms are gastrointestinal bleeding (GIB) and non-specific abdominal pain [1,2]. GISTs are usually categorized into very low, low, intermediate and high risk potential [1]. However, GISTs have different clinical, histological and immunohistochemical features depending upon tumor location which is an independent risk factor for tumor recurrence. The main aim of surgical treatment is complete removal of the tumor (en bloc) with a negative surgical margin. Surgical approach depends upon tumor location and size and varies from local excision to a pancreaticoduodenectomy [1,2].

The following paper presents a case of DGIST from teaching university hospital.

This work has been reported in line with the SCARE criteria [3].

## 2 Presentation of case

75 years old Caucasian male, smoker, with history of excessive drinking, was seen at Internal medicine Emergency department due to hematemesis, hemohesia and dizziness. Few days before he felt weak. He had history of gastritis, colon polyps resection, arterial hypertension, chronic obstructive pulmonary disease and depression. Family history was positive for breast and skin cancer. Upon admission on emergency department blood pressure was stable, he was tachycardic, skin was pale. There was slight pain in epigastrium, without signs of peritoneal irritation. Digital rectal examination was within normal.

**Fig. 1:**
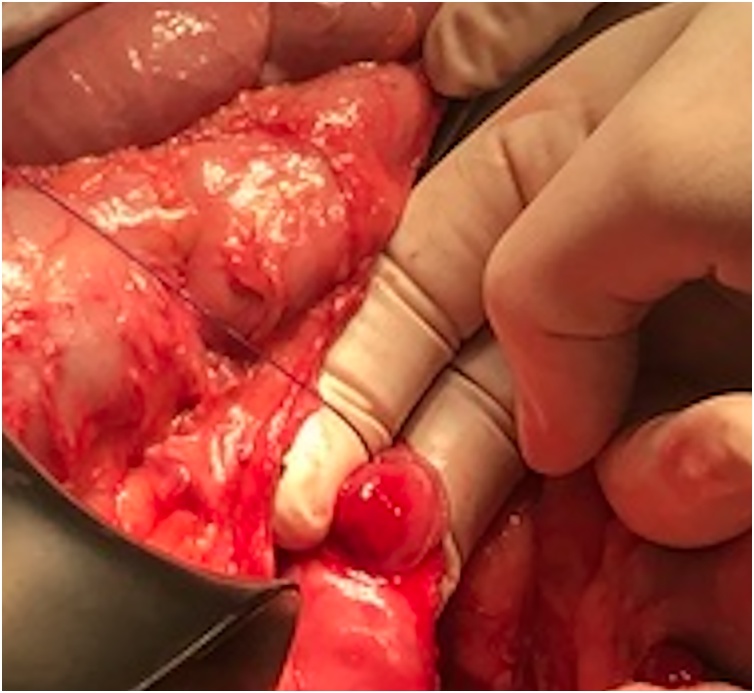
Intraluminal tumor with central bleeding ulceration.

**Fig. 2:**
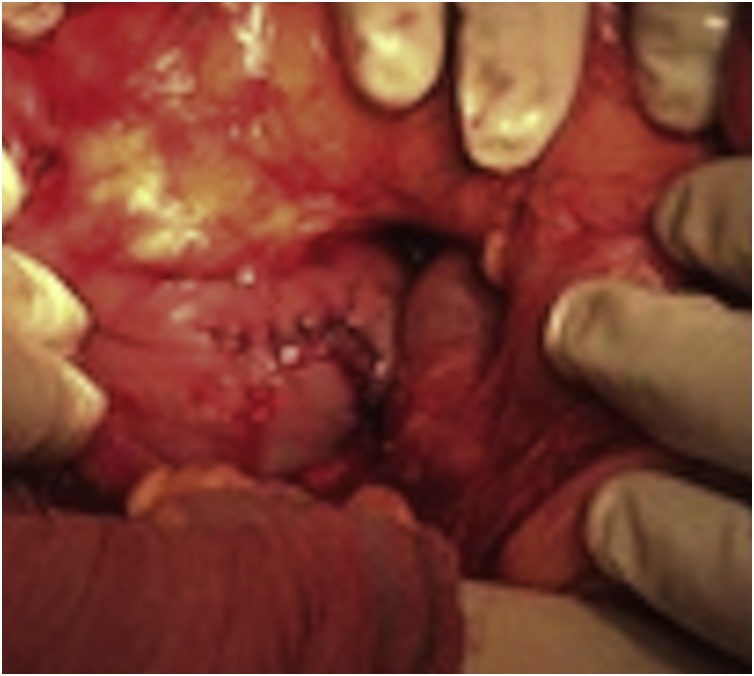
Repair of the duodenotomy with interrupted sutures.

**Fig. 3:**
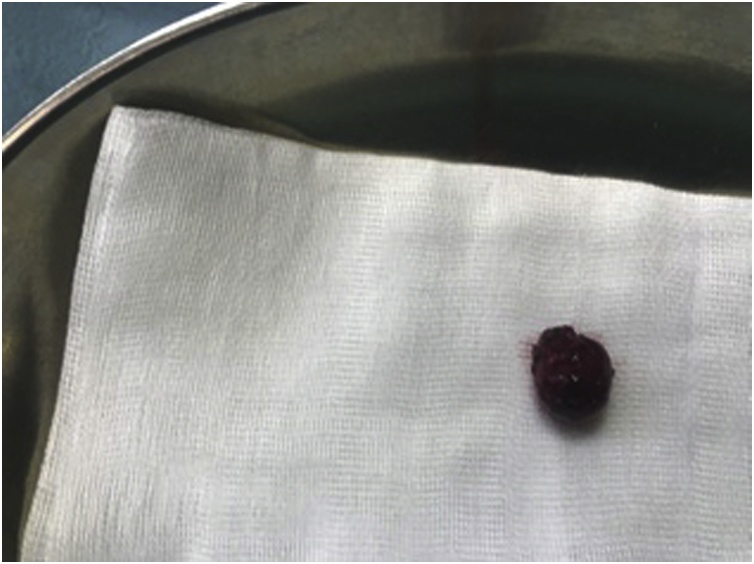
Tumor after excision.

Due to GIB and anemia, he was admitted to the Department of gastroenterology. On the admission day hemoglobin (Hb) level was 71 g/dL and an emergency oesophagogastroduodenoscopy (EGD) was performed which showed some hematous content in the stomach without clear source of active bleeding being found. He received transfusion of 3 units of red blood cells (RBC). Patients’ condition was worsening, he lost consciousness hence the EGD was repeated. In the third portion of duodenum a submucous, actively bleeding tumor was found. Endoscopic hemostasis was unsuccessful and patient was in haemorrhagic shock. He was transfered in the operating room for an emergency explorative laparotomy. Procedure was performed by two experienced abdominal surgeons (more than 10 years experience in abdominal surgery; one of the surgeons has title of university professor). After extensive mobilization of the duodenum a 2 × 2 cm intraluminal tumor with central bleeding was found in the third portion of the duodenum (Fig. 1). The diseased part of the duodenum was excised (Fig. 2) and the duodenotomy was closed with interrupted sutures (Fig. 3). During surgical procedure he received 8 units of RBC, 4 units of FFP (fresh frozen plasma) and 1 unit of concentrated thrombocytes. Postoperative period was uneventful, only small inflammation of the laparotomy was present which was cured with conservative measures. Hb level was stable. Patient was discharged on the 9th postoperative day. Pathologist reported spindle cell GIST, without mitosis, low grade, pT1 R0. Patient was discussed at the multidisciplinary team meeting (MDT) which decided that only a regular follow-up was indicated.

## 3 Discussion

GISTs present approximately 1% of the gastrointestinal tumors [4]. Estimated incidence in adult population is 10–20/1000000 [4,5]. Average age at presentation of GIST is 50–65 years. In population under 30 years incidence is low (<5%) [5,6,8]. In our case, the age of onset was higher than average.

GISTs are most common mesenchymal neoplasms and typically are subepithelial [1,2]. Most commonly they originate from the interstitial cells of Cajal in the muscularis propria. Predilection sites are stomach and small intestine [2]. All GIST are potentially malignant even though they can have benign appearance [6]. Duodenal GIST are rare [1,7]. Median size of DGIST lesion is about 4 cm [6]. In our case, tumor was smaller than the average. Our patient had risk factors for upper gastrointestinal bleeding - alcohol abuse and gastritis. Considering DGISTs are relatively rare, EGD diagnosis of DGIST was challenging.

Leading symptoms are nonspecific abdominal pain, GIB (melena, hematemesis, hematochezia), and symptomatic anemia [1,7]. Clinical, histological as well as immunohistochemical features vary, depending on tumor location [1]. Diagnosis is histologically confirmed by expression of positive immunohistochemical staining for CD117, which is present in 95% of cases while CD34 stains positive in 70% of GIST [4]. EGD is usually useful in detecting DGIST [6]. Endoscopic ultrasound is helpful in diagnostics and determining location from where the lesion arises (submucosal, intramural, extramural) (6, 8). CT and MRI are standard diagnostic modalities for estimating the primary lesion and detecting possible distant disease [6]. Better prognosis is expected in patients with smaller tumors and early clinical symptoms [1]. Prognostic factors for GIST are classified into *tumor related* (anatomic site, histologic type, size of tumor, depth of invasion, grade, M category, mitotic rate, presence of KIT mutation, mutation site in KIT or PDGFRA gene, surgical resection margins, presentation status, tumor hypoxia, Ki-67, TP53), *host related* (NF1, age) and *environment related* (quality of surgery) (8). DGIST recurrence depends more on tumor biology than surgical approach/type of surgical procedure or microscopic margins [1]. Rate of recurrence and metastases is predicted by estimating tumor diameter and mitotic ratio [6].

Treatment should be multidisciplinary involving medical oncologist, gastroenterologist, surgeon, pathologist, radiologist, nuclear medicine specialist etc. Such patients should be treated in a reference centre [8]. Our patient was first admitted at the Department of gastroenterology and then at the Department of Abdominal surgery, in teaching university hospital. After receiving pathohistological report, patient was presented at MDT, which consist of abdominal surgeon, gastroenterologist and medical oncologist, and decision about regular follow-up was made.

Surgical treatment is a golden standard, although surgical approach varies depending on tumor size, location and invasion into adjacent organs (stage of disease) (1, 8). Preoperative radiological staging (CT/MRI) is of crucial importance for surgical procedure planning [8]. In cases such as in ours when massive bleeding is present, lifesaving surgical procedure has priority over other diagnostic modalities.

Standard treatment of localised DGIST is complete surgical excision (en bloc) with negative surgical margins, without lymphadenectomy of clinically negative lymph nodes [[6], [7], [8]]. In case of involvement of D2 or larger tumors, probability of undergoing pancreaticoduodenectomy is higher [1]. Lymphadenectomy is still a controversial issue in surgical treatment of DGIST [7]. In our case explorative laparotomy with extensive duodenal mobilization and duodenotomy of D3 was performed, due to challenging tumor localization. As the tumor was small segmental resection of duodenum was possible. Mokhtare et al. reported a case of acute bleeding from tumor located in D3 that was first managed successfully with endoscopic procedure, later surgical removal of tumor was performed [6]. Valli et al. also reported a case of acute bleeding from tumor near major duodenal papila, which was successfully managed endoscopically with clips and epinephrine injection, after radiological staging surgical removal was performed [4]. In cases when endoscopic hemostasis is possible, it’s highly recommended. After endoscopic procedure additional radiological imaging is performed for staging purposes and optimal surgical treatment is performed.

In cases of minimally invasive approach (laparoscopic or robotic), principles of oncologic surgery should be implemented [1,8]. Radio- and chemotherapy are indicated in palliative cases. Immunotherapy with imatinib mesylate is indicated as a neoadjuvant therapy of GIST or in patients with recurrent disease [6,8].

## 4 Conclusion

DGIST may be source of life threatening bleeding. In such cases surgical treatment has priority over diagnostics. Aim of surgical treatment is to remove tumor en bloc, following the principles of oncologic surgery. Surgical approach depends upon tumor location and size. Recurrence rate depends upon tumor location and extend of removal. Because of the rarity, role of lymphadenectomy and most appropriate surgical approach are unclear.

## Conflicts of interest

All authors have no conflict of interest to disclose.

## Funding

Authors did not receive any form of funding.

## Ethical approval

Patient was treated according to current guidelines. Ethical approval is not required.

## Consent

Written informed consent was obtained from the patient for publication of this case report and accompanying images. A copy of the written consent is available for review by the Editor-in-Chief of this journal on request.

## Author contribution

Milena Taskovska - preparation of manuscript, literature review

Mirko Omejc - surgical procedure, review of the manuscript

Jan Grosek - surgical procedure, literature revire, review of the manuscript

## Registration of research studies

Not applicable.

## Guarantor

All authors accept full responsibility for case report presented.

## Provenance and peer review

Not commissioned, externally peer-reviewed.
